# Nanocommunication design in graduate-level education and research training programs at Osaka University

**DOI:** 10.1007/s11051-014-2595-8

**Published:** 2014-08-12

**Authors:** Mizuki Sekiya, SoonHwa An, Masafumi Ata

**Affiliations:** Office of R&D Strategy, Nanosystem Research Institute of National Institute of Advanced Industrial Science and Technology (AIST), Tsukuba, Japan

**Keywords:** Public engagement, Education and capacity building, Technology governance, R&D strategy, Nanotechnology

## Abstract

After more than ten years of strategic investment research and development supported by government policies on science and technology, nanotechnology in Japan is making a transition from the knowledge creation stage of exploratory research to the stage of making the outcomes available for the benefit of society as a whole. Osaka University has been proactive in discussions about the relationship between nanotechnology and society as part of graduate and continuing education programs. These programs are intended to fulfill the social accountability obligation of scientists and corporations involved in R&D, and to deepen their understanding of the relationship between science and society. To meet those aims, the program has covered themes relating to overall public engagement relating to nanotechnology governance, such as risk management of nanomaterials, international standardization for nanotechnology, nanomeasurement, intellectual property management in an open innovation environment, and interactive communication with society. Nanotechnology is an emerging field of science and technology. This paper reports and comments on initiatives for public engagement on nanotechnology at Osaka University’s Institute for NanoScience Design, which aims to create new technologies based on nanotechnology that can help realize a sustainable society.

## Creating new relationships between science and society

During the twentieth century, referred to by some as the century of science, industrial development which brought dramatic changes to the human environment and societal systems was supported by developments in individual disciplines such as physics, chemistry, biology, medicine, pharmaceutical sciences, mechanical engineering, and electronic engineering. For example, the development of the chemical industry was supported by new discoveries and knowledge creation in the discipline of chemistry. People involved in R&D of science and technology generally believe that activities relating to new discoveries and knowledge creation should be free and independent from society. Researchers involved in R&D in science and technology generally focused only on R&D, and left the application of new discoveries and knowledge to the players who applied and used them. Many of those involved in R&D generally felt no responsibility for the application of their findings in society.

The industrial structure that developed under these frameworks and ways of thinking has given rise to a host of risks, from industrial accidents and pollution at the local level to problems at the global scale, like environmental destruction and climate change. By the end of the last century, various crises that had erupted as a result of science and technology led to a loss of public trust and support, and an awareness gap grew between society and those involved in R&D. In this chaotic situation, people began to reconsider the role of science and technology, and the search began for a new relationship between science and society. One conclusion of the scientific community has been that there is a need to strengthen the relationship between science and society.

From June 26 to July 1, 1999, just before the dawn of the twenty-first century, the United Nations Educational, Scientific and Cultural Organization (UNESCO), and the International Council for Science (ICSU) held a World Science Conference in Budapest, the capital of Hungary. Participants specializing in a variety of disciplines debated the optimal relationship between science and society if we are to create a sustainable society. The Declaration on Science and the Use of Scientific Knowledge (Budapest Declaration) adopted by the conference includes the three traditional objectives of science for knowledge, science for peace, and science for development and adds a new one: science in society and science for society (UNESCO/ICSU 1999).

Scientists until then had a tendency to see science and technology as their domain and to see no need for the involvement of non-specialists. Meanwhile, the public in general did not see the need (or believe it had the ability) to debate or comment on how science should be, or to affect policy relating to science and technology. Due to the greater prominence of risks arising from science, there was also an increasing awareness that not only scientists but also society as a whole should participate actively in discussions about science. The Budapest Declaration declares that scientific knowledge must serve society and reflects a major shift in awareness from the traditional view that science should be separate from and not affected by society. It presents a specific new purpose for science and represents a turning point, encouraging the fostering of a new relationship between science and society.

## Practical efforts to create a new relationship between science and society

The principles underlying the Budapest Declaration, fostering a new relationship between science and society, began to have an impact on science policy in individual countries. In Japan, for example, the Second Science and Technology Basic Plan (adopted by Cabinet decision on March 31, 2001, with implementation beginning April 1) included the words, “Keeping in mind the idea of science and technology in and for society, it is indispensable to establish fundamentals of interactive communication from science and technology and society” (MEXT 2001). Thus, the concept of science existing for society was written into policy, but the concept was not put into practice during implementation of the Plan, due to an absence of concrete discussion about actions under the strategies for each of the eight R&D areas covered by the plan. In the Second Science and Technology Basic Plan, nanotechnology was defined under the heading of “nanotechnology and materials science,” and major R&D funding began in these areas, but the concept of science existing for society was not actually put into practice.

Recognizing the need to put the concept of science and technology in and for society into practice, as discussions began in fiscal 2005 on the directions for the next Basic Plan, we at the National Institute of Advanced Industrial Science and Technology (AIST) joined with the National Institute for Materials Science (NIMS), National Institute for Environmental Studies (NIES), National Institute of Health Sciences (NIHS), and others to conduct a research project aiming to develop policy proposals for activities on the societal implications of nanotechnology. Based on a year of studies, the research project proposed concrete initiatives that should be implemented by the government to create a new relationship between science and society (MEXT 2005). The Third Science and Technology Basic Plan reflected these recommendations and carried more specific language than the abstract wording in the second plan regarding what the government should do. Regarding a nanotechnology and materials science strategy, the new plan clearly mentions such topics as nanomaterial risk management, research into environmental health and safety (EHS), and international standardization (MEXT 2006).

As implementation of Japan’s Third Science and Technology Basic Plan began, serious efforts were also initiated within international frameworks like the International Organization for Standardization (ISO) and Organization for Economic Cooperation and Development (OECD), relating to international standardization for nanotechnology and development of policies to manage nanomaterials, etc. Japan participated actively in these international frameworks, and puts real effort into areas such as EHS research for nanomaterials, formulation of risk management policies, and international standardization of nanotechnologies. These initiatives for public engagement and nanotechnology were concrete actions based on government policy during the five-year implementation of the Third Science and Technology Basic Plan, which started in fiscal 2006.

Implementation of the Fourth Science and Technology Basic Plan began in August 2011, after revisions were made to incorporate responses to the Great East Japan Earthquake that hits the country on March 11 of that year. In that plan, the theme of recovery and reconstruction after that disaster was pushed to the fore. The plan also incorporated a major departure from the previous R&D strategies for specific priority fields, instead moving toward problem-solving strategies such as green innovation and life sciences innovation. Also, science/technology policies and innovation policies, which had previously been independent of each other, were grouped together, based on the approach that more integration of the two was needed. Reflecting the spirit of the Budapest Declaration, the plan also emphasized the importance of science in society. The government went one step further than in previous Science and Technology Basic Plans by articulating the need for public accountability and emphasizing the public nature of science and technology policy. The plan also says that to address ethical, legal, and social issues (ELSI) relating to science and technology, the government will show leadership and engage in outreach activities such as interactive communication with the public (MEXT 2011). The challenge is finding concrete ways to do so.

## Nanotechnology R&D and public engagement

**Fig. 1:**
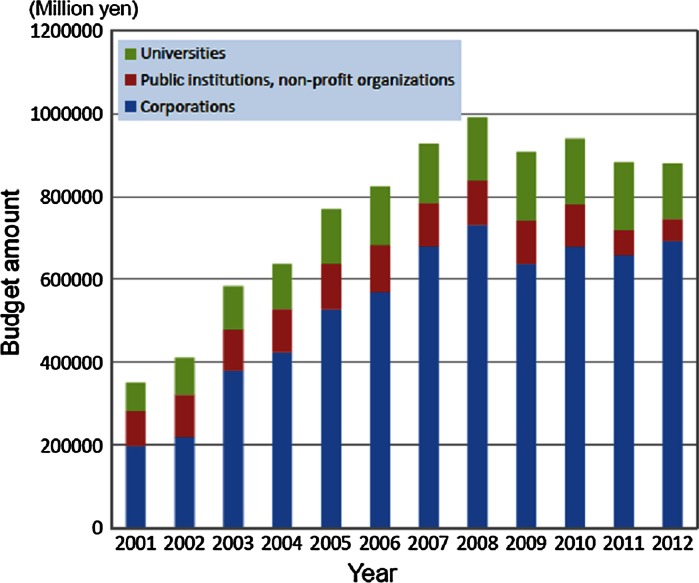
Total budget for nanotechnology and materials in the government’s Second and Third Science and Technology Basic Plans (MIC 2012)

Figure 1 shows how Japan invested strategic resources into nanotechnology R&D over the ten-year period covered by the Second and Third Science and Technology Basic Plans (MIC 2012). It is worth noting that the Japanese private sector’s investment into nanotechnology R&D during this period was far greater than that of the government.

Nanotechnology had two new dimensions not previously evident in research and development for science and technology. One was that nanotechnology had become interdisciplinary, integrated in an overarching way across numerous disciplines. The context for this was the need for nanotechnology R&D to create nanosystems based on the fusion of top-down nanotechnologies (made possible with the arrival of the ultimate precision equipment manufacturing technologies) and bottom-up nanotechnologies (technologies that can organize and assemble individual atoms). Also, cross-disciplinary integration and industry-academia-government collaboration in R&D frameworks were also being considered essential for the advance of multidisciplinary R&D, and the government also actively supported collaborative approaches.

The other dimension, as an extension of the concepts of the Budapest Declaration, is the elucidation of the diverse impacts of technology on society, and the development of activities relating to societal implications to feed back into R&D. Because the societal implications include socioeconomic impacts, nanotechnology work expanded to cover interdisciplinary R&D that included sociology and economics.

There are also many nanotechnology R&D topics with these kinds of characteristics that need to be advanced based on society’s trust, and topics that must be tackled in order to appropriately apply and utilize the findings in society. More specifically, these diverse and varied issues range from the problems of environment, health and safety (EHS) of nanomaterials, international standardization of nanomaterials, and issues that directly affect international competitiveness such as intellectual property management of nanotechnology, to issues like science communication and outreach activities. It would not be an exaggeration to state that all issues about public engagement on nanotechnology, besides relating to R&D for core nanotech technologies, are actually at the very interface between nanotechnology R&D and society.

## Current status of public engagement education at university in Japan

To ensure that concrete efforts continue to address topics of public engagement such as risk management and international standardization on which work had already begun based on the government’s science policies, it is essential to bring public engagement topics into educational and capacity building fora. If initiatives are taken in the form of university educational and capacity building programs, public engagement efforts can be also expected to become established in new science/technology disciplines yet to develop through interdisciplinary approaches in the future. As described above, nanotechnology has developed as a multidisciplinary field. It has become commonly understood that R&D outcomes will not lead to innovation unless the issues of public engagement are addressed comprehensively, with cross-disciplinary integration and industry-academia-government collaboration. Such issues include risk assessment and risk management, international standardization, intellectual property management, science communication, and outreach activities. Efforts addressing these issues require educational and capacity building programs accessible for any students aiming at careers not only in science and technology but also in economics, sociology, and other areas.

Despite this, it was rare to find cases in which the issues of public engagement were actually covered by university education in areas like nanotechnology and sociology in Japan. Programs were being offered to provide university students basic knowledge about nanotechnology, but it is fair to say that no university addressed issues of public engagement as a part of that education. If engineering students only gain basic knowledge about nanotechnology, they will start their careers without any knowledge about matters such as how that technology is applied in society, what kinds of issues arise, and how they are being addressed. Is it not important for students who aspire to a career in nanotechnology R&D to know about the Budapest Declaration? Or about risk assessment and risk management? Or business strategies for intellectual property, or international standardization in an open innovation environment?

If these things are important, why not offer multidisciplinary educational and capacity building programs on these topics at the university level? As stated above, during the last century, the tendency was for each individual academic discipline to be supported by one particular industry. The industrial structure had developed this way, and became reflected in university education, particularly in science-related faculties. Educational programs were designed with the mission of creating specialists who had concentrated on learning specialized information. By the twenty-first century, the cumulative body of knowledge in science and technology had expanded dramatically, bringing the end to an era in which an individual industry supported an individual discipline. Despite this, Japanese university engineering and science faculties still today retain the vestiges of the older scientific framework, with departments of physics, chemistry, biology, and also physical engineering, chemical engineering, mechanical engineering, and so on. One could hypothesize that the reason why multidisciplinary initiatives on public engagement relating to nanotechnology do not take root is that educational frameworks at universities are unable to adapt to the new need for interdisciplinary education. The old characteristics persist.

The old habits were overcome outside of Japan, and the educational setting for public engagement in nanotechnology in the United States and Europe differs from Japan (Jones et al. 2014; Guston 2011, 2014; Flipse et al. 2012). In the United States, National Science Foundation-supported education and collaboration network for public engagement in nanotechnology are created among universities and institutions. Useful as well as practical programs including educational tools are provided at universities such as the Center for Nanotechnology in Society at Arizona State University, the Center for Nanotechnology in Society at University of California, Santa Barbara, University of Wisconsin-Madison, University of Washington etc. (CNS-ASU 2014; CNS-UCSB 2014; University of Wisconsin-Madison 2014; University of Washington 2014). In Europe, there are a lot of programs on education and capacity building, which are related to public engagement in nanotechnology R&D such as Nanosmile, etc., through Framework Program (Nanosmile 2014).

## Initiatives at Osaka University for public engagement on nanotechnology

In this context, Osaka University became the first Japanese university to actively tackle the topic of public engagement relating to nanotechnology within the educational offerings for science and engineering graduate students and in continuing education programs for people already in the working world. The Institute for NanoScience Design (INSD) at Osaka University took the leading role in these important programs. This initiative is called the “Public Engagement and Nanotechnology,” under Osaka University’s Nanotechnology Advanced Interdisciplinary Education, Research, and Training Program.

**Fig. 2:**
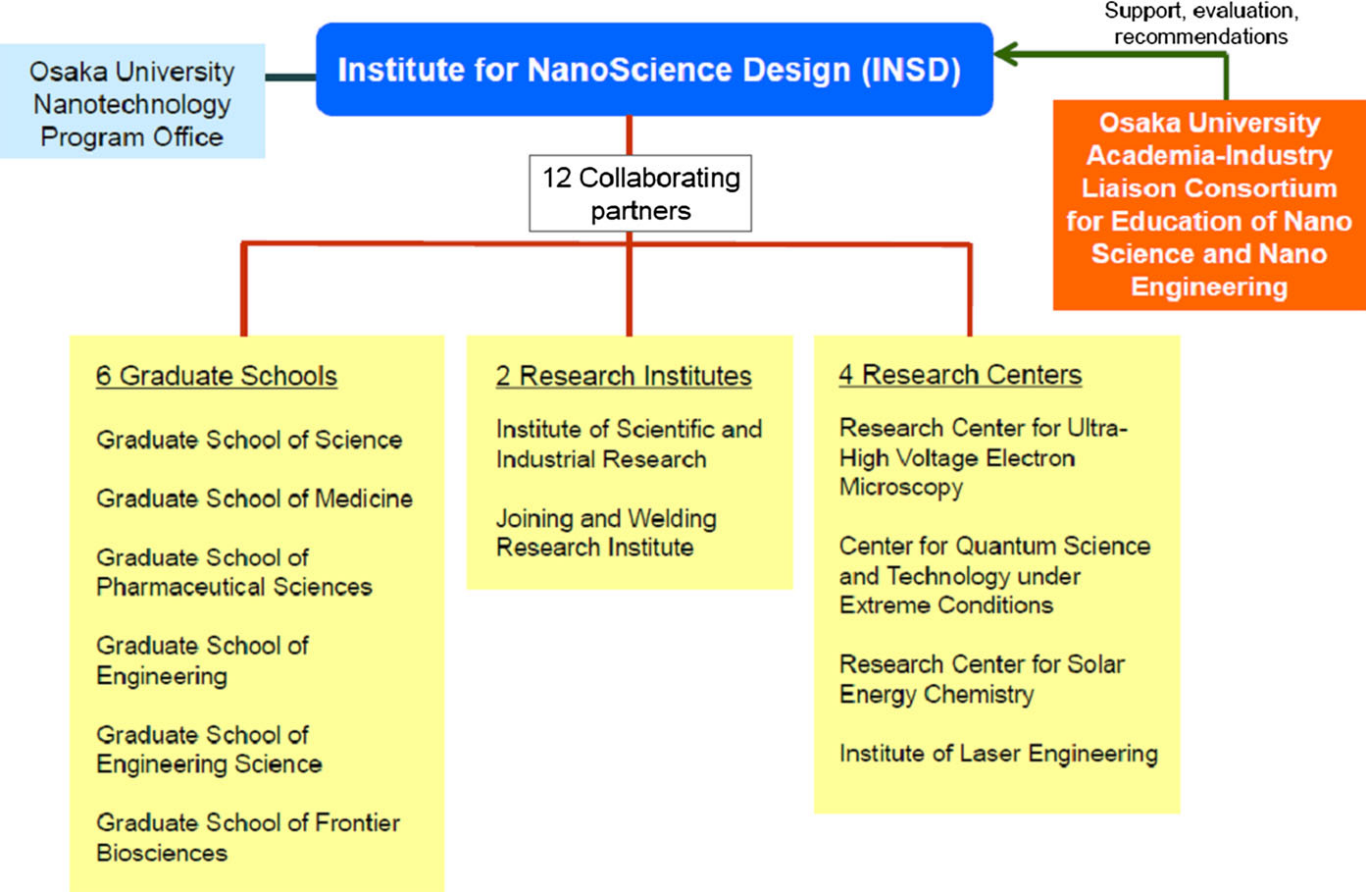
Organizational chart of the Institute for NanoScience Design (INSD 2012)

INSD was launched on December 1, 2008 with the aim of fostering highly trained human resources in Japanese manufacturing, through collaboration among the related departments at Osaka University, and also with industry-academic collaboration, and collaboration with other universities in Japan and overseas. The institute aims to create an interdisciplinary minor degree with a practical emphasis, to foster integrated design capacity on nanoscience, at the graduate level, and it targets both graduate students and working people. The capacity building program plays a coordinating role, making use of nanotechnology-related departments and human resources at the university, including six graduate schools, two research institutes, and four research centers (INSD 2012; Itoh et al. 2009, 2010; Itoh 2011a, b) (Fig. 2).

INSD started with two pilot initiatives on the theme of public engagement and nanotechnology in fiscal 2009, and then launched a full lecture program in fiscal 2010.

One of the pilot programs in fiscal 2009 was an Introductory Seminar Series on Public Engagement and Nanotechnology, held jointly with Osaka University’s Academia-Industry Liaison Consortium for Education of NanoScience and NanoEngineering. Another pilot program was part of the seminar series entitled “Nanotech Seminar 2009 - Risk Management Approaches,” conducted jointly with AIST. This program was part of a fiscal 2009 project by AIST, entitled “Development of comprehensive database index for a basis of facilitation of nanotechnology R&D,” and it was a complementary research project for a coordination program of the Council for Science and Technology Policy (CSTP, a Cabinet level advisory body), entitled “Developing Nanotechnologies and Engaging the Public.” Both seminars were open to not only students but also to the public; anyone could participate, including people from the business world and research institutes (MEXT 2009; AIST 2010). Reviewing the outcomes of the pilot programs, INSD found that these programs for the education of R&D personnel would promote researchers’ awareness of public engagement issues relating to nanotechnology and help them consider the issues as a part of their own research. It concluded that these results were valuable and deserved further effort. As a result, INSD decided to continue. Thus, it offered a Saturday intensive lecture series entitled “Special Lectures on Public Engagement and Nanotechnology” in fiscal 2010. Since fiscal 2011, students can earn academic credits by participating in the lectures (Table 1).

**Table 1:** Programs and pilot programs

Year	Dates	Program title	Organizer
FY2009	August 21, 2009	Introductory Seminar Series on Public Engagement and Nanotechnology	Osaka University Academia-Industry Liaison Consortium for Education of NanoScience and NanoEngineering Institute for NanoScience Design, Osaka University
FY2009	October 14, 2009	Nanotech Seminar 2009 – Risk Management Approaches	AIST Institute for NanoScience Design, Osaka University
FY2010	June 5, June 19, 2010	Saturday intensive series “Lectures on Public Engagement and Nanotechnology – A”	Institute for NanoScience Design, Osaka University
FY2011	May 28, June 18, July 2, 2011	Saturday intensive series “Lectures on Public Engagement and Nanotechnology – A”	Institute for NanoScience Design, Osaka University
FY2012	June 2, June 30, July 14, 2012	Saturday intensive series “Lectures on Public Engagement and Nanotechnology – A”	Institute for NanoScience Design, Osaka University
FY2013	May 18, June 15, July 6, 2013	Saturday intensive series “Lectures on Public Engagement and Nanotechnology – A”	Institute for NanoScience Design, Osaka University

**Fig. 3:**
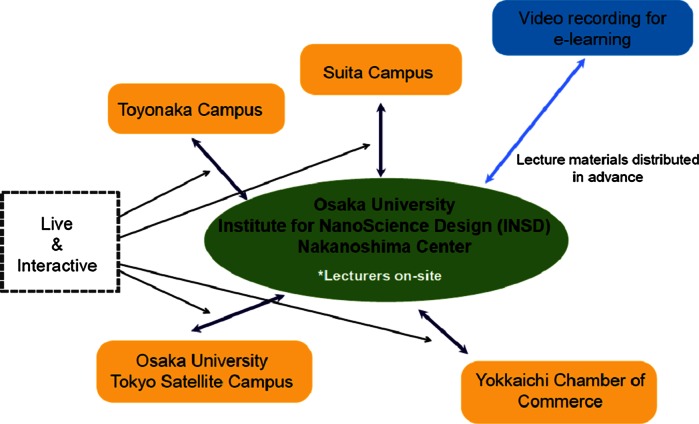
Participation in classes facilitated by remote lecture sharing system (INSD 2012)

The involvement of Osaka University’s Academia-Industry Liaison Consortium for Education of NanoScience and NanoEngineering gives special status to this program. The participation of working people adds value to the content of the Saturday intensive lecture series, and the involvement of the Consortium (with members coming from corporations in the region) is an important pillar of the program. In the context of issues about public engagement relating to nanotechnology, INSD provides a forum for people to acquire knowledge and learn systematically about nanoscience and nanoengineering research. The Saturday Special Lectures on Public Engagement and Nanotechnology provide a place for participants to learn about the relationship between society and nanoscience/nanotechnology research. The Saturday lectures have some unique features. One feature is that participants are not just listening to the lectures, but also have the opportunity for in-depth discussion about the content, including through a question and answer session. Each lecture is ninety minutes long, including the Q&A session, and an extra ninety minutes is set aside for discussion. Another feature is, as mentioned above, participation from the working world. Participants include not only graduate students from various undergraduate departments and graduate schools, but also working persons involved in corporate R&D or practitioners in chemicals management, etc. All participate on an equal footing in the lectures, resulting in benefits for both sides. To support this style of lecture, the Institute uses a remote lecture system (Itoh et al. 2009, 2010; Itoh 2011a, b). The system links the Nakanoshima Center of Osaka University with campuses in Suita and Toyonaka, as well as venues in Yokkaichi and Tokyo. The other venues are connected live, in real time, with the Nakanoshima venue for all parts of the lecture, including the Q&A session. During the discussion after the main lecture, each venue is still connected with Nakanoshima venue, so participants can participate in discussions even if they are distant from the main venue (Fig. 3).

## Discussions at the Special Lectures on Public Engagement and Nanotechnology

**Fig. 4:**
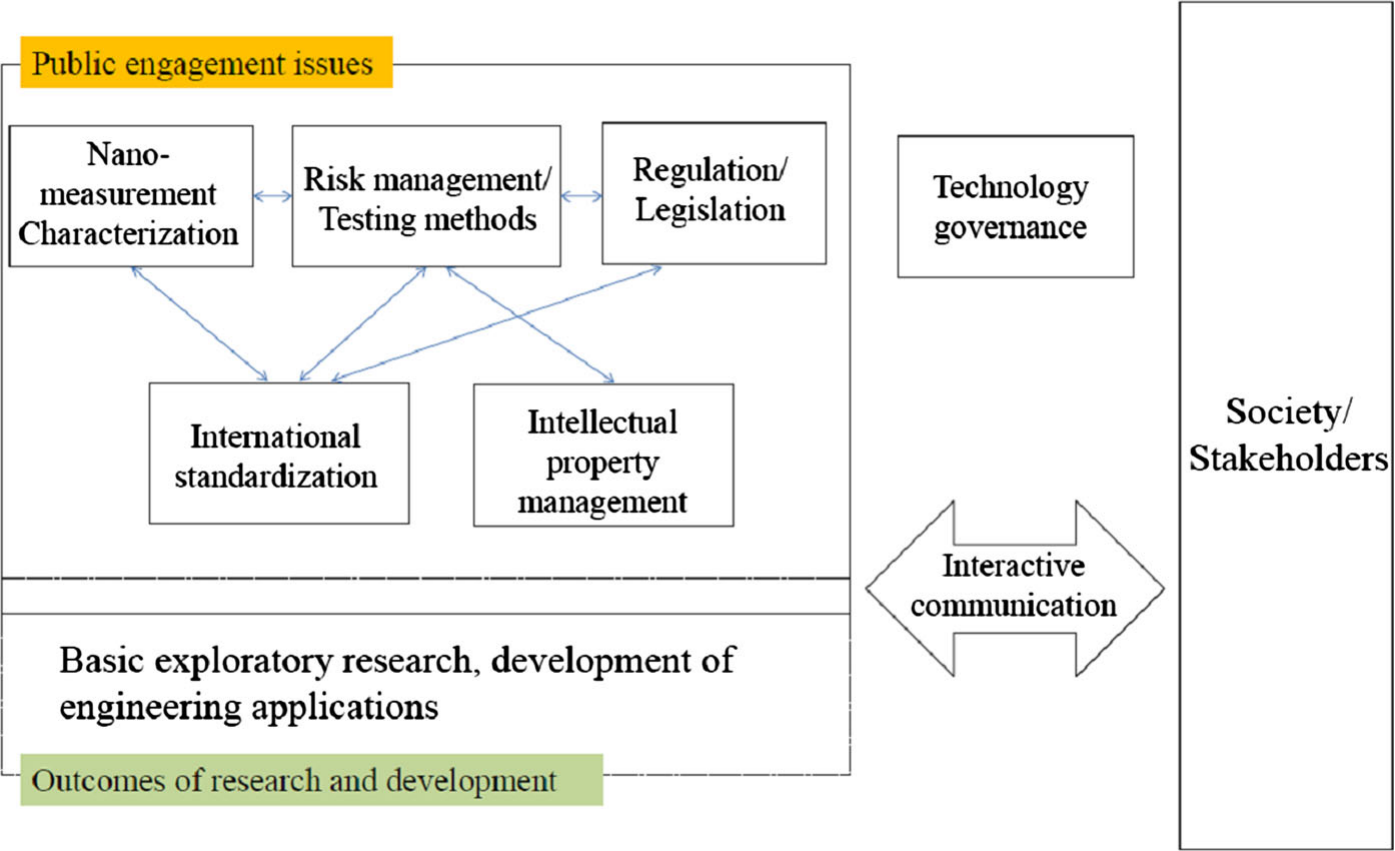
Key issues for public engagement and nanotechnology, and their relationship with society

None of the issues about public engagement relating to nanotechnology can be addressed in isolation. The development of measurement technologies is critical for EHS research. EHS research findings form the basis for formulation of regulatory policies. Meanwhile, international standardization provides evaluation criteria for regulation, and advances in measurement technologies intimately affect international standardization. One can see that EHS research and international standardization, which at first glance may appear to be unrelated, are also profoundly linked. Regulatory policies are the rules of business, and at the same time are an important key to international standardization to promote the smooth and fair conduct of business. Topics like international standardization and intellectual property management that at first may seem to be have a tension between them but closely interlinked in the sense that they create new rules for business. None of the individual issues are more important than others, and in fact, each one is mutually related in an organic way. Regarding the Special Lectures on Public Engagement and Nanotechnology, the various topics are organized to facilitate systematic and synergistic learning, even for people who are new to the topics (Fig. 4).

The Special Lectures were held three times, taking up a full day on three separate Saturdays. From the first to the third times, lectures and discussion were presented by two lecturers each time, and on the final day, a panel discussion was held with the participation of all of the lecturers. In fiscal 2012, about 40 graduate students and 40 working people participated. In fiscal 2013, 35 graduate students and 35 working people participated (Table 2).

**Table 2:** Lectures in fiscal 2012 and 2013

Date	Lecture topic	Description
June 2, 2012 (Sat.) morning	Introduction	Lecture series guidance
June 2, 2012 (Sat.) morning	What is public engagement on nanotechnology?	Overview from various angles on trends relating to R&D and current status of public engagement, from the perspective of an early advocate and promoter of public engagement on nanotechnology in Japan
June 2, 2012 (Sat.) afternoon	Intellectual property management of nanotechnology	Intellectual property strategy for nanotechnology, new business, public engagement, and patent strategy. Consideration of what nano venture entrepreneurs need to do
June 30, 2012 (Sat.) morning	International standardization of nanotechnology measurement	History and significance of characterization of nanomaterials; standardization trends and issues for nanomaterial analysis for surface and nano dimensional analytical methods
June 30, 2012 (Sat.) afternoon	Nano risk and risk management	How to view and deal with nano risk, with cases from Japan and overseas
July 14, 2012 (Sat.) morning	International standardization of nanotechnology	Standardization/regulatory trends relating to nanomaterial definition, product labeling, and safety; related industry initiatives overseas; what Japanese corporations should do
July 14, 2012 (Sat.) afternoon	Making use of nanotechnology information	Effectiveness of information use in R&D, through PEN and other activities that are tools of technological governance
July 14, 2012 (Sat.) afternoon	Panel discussion	Panel discussion with lecturers from 1st to 3rd days
May 18, 2013 (Sat.) morning	Introduction	Lecture series guidance
May 18, 2013 (Sat.) morning	Public engagement on nanotechnology	Public engagement issues that must be addressed when nanotechnology R&D moves ahead even before social infrastructure is in place for management; overview from lecturer’s personal experience
May 18, 2013 (Sat.) afternoon	Science and technology communications	Overview of concepts and history of risk communication and science communication, and discussion of changing relationship between science/technology and society as the context for the emergence of risk/science communication
June 15, 2013 (Sat.) morning	International standardization of nanotechnology	International standardization activities with optical catalysts as case study; development trends of optical catalysts; certification programs by optical catalyst industry; what is needed to create a constructive relationship between emerging S&T and society
June 15, 2013 (Sat.) afternoon	Nanotechnology intellectual property	Importance of intellectual property in materials development; how to secure and utilize strong property rights; desired state of intellectual property in the era of open innovation with Osaka University’s intellectual property activities as case study
July 6, 2013 (Sat.) morning	Nanomaterial risk assessment	Research on nanomaterial safety; overview of importance of research for design of safe nanomaterials; future issues for ongoing future development of nanotechnology
July 6, 2013 (Sat.) afternoon	Making use of nanotechnology information	Discussion on desired responses to societal needs, on the front lines of R&D of nanotechnology, an emerging science and technology; introduction of PEN and PENGIN interactive communication tools
July 6, 2013 (Sat.) afternoon	Panel discussion	Panel discussion with lecturers from 1st to 3rd day

The topic of intellectual property management is one example of the way in which INSD designed the lectures to facilitate overarching and systematic understanding of the various issues about public engagement and nanotechnology. If students who have studied nanotechnology end up employed with a company, it is likely they will have opportunities to learn how to complete documentation on intellectual property as a part of training in the workplace. However, in an open innovation environment, they would have almost no opportunity at the company to learn about cases of how to utilize intellectual property and standardization and spread the technology, or how to secure profits. This environment has globalized the supply chain and rapidly commoditized technologies. If one is to provide nanotechnology R&D outcomes to society, it is essential to consciously address issues of intellectual property management as well as other public engagement issues.

Generally, intellectual property is a framework to obtain profit by exercising exclusive control of a technology. In other words, it is a closed innovation tool. On the other hand, standardization, because it rapidly spreads a technology and does not make it exclusive, demonstrates its potential in an open innovation environment, because it helps the technology spread rapidly. Seen this way, intellectual property in a closed innovation environment may appear to have no potential to be utilized in an open innovation environment. The challenge today, however, is to move forward from that point and skillfully use intellectual property of a closed nature in an open innovation environment. In other words, the challenge is to seek successful management approaches. As solutions to these kinds of challenges, the fiscal 2012 lectures introduced initiatives like yet2.com, which connects technologies and market opportunities. The Osaka University lecturers also made a special effort to address public engagement issues such as these ones that are important for the business world.

## The significance of nanotechnology and public engagement efforts at Osaka University

The Special Lectures on Public Engagement and Nanotechnology exposed many participants, particularly graduate students, to a large amount of new terminology. Many participants, however, expressed the positive view that they had benefited from being able to think in their own terms in the free discussions after each lecture, by being exposed to new perspectives on nanotechnology. Also, new ideas that arose from participants during the featured discussions after the lectures added to the value of the discussions. Several of the lecturers, specialists in their fields, commented that they were impressed by the ideas that arose.

At the free discussions, Tadashi Ito and other instructors from INSD served as moderators, separating the participants into groups, with an effort to balance the numbers of working people, and undergraduate and graduate students. Initially, the small group discussions on unfamiliar topics of nanotechnology public engagement may have been slow to begin, as they were being conducted in a style somewhat different from regular university classes. Skillful moderation by instructors and lecturers, however, combined with participants’ keen interest, made the time go quickly. The benefits of the discussions after the lectures also included the fact that students who found the new experience to be challenging could learn from the example of participants from outside the university who were more accustomed to these types of discussions. Currently, Osaka University is the only university in Japan that offers classes where students can gain academic credits from lectures on nanotechnology and public engagement. Also, undergraduate students generally have very few opportunities to learn together with working people, and to have a discussion with them and exchange opinions and information regarding science, technology, and society. This program could certainly be seen as a valuable and concrete effort to demonstrate the concept of science in society, an important theme in Japan’s Fourth Science and Technology Basic Plan.

## Holding the key to international competitiveness: public engagement in science education

**Fig. 5:**
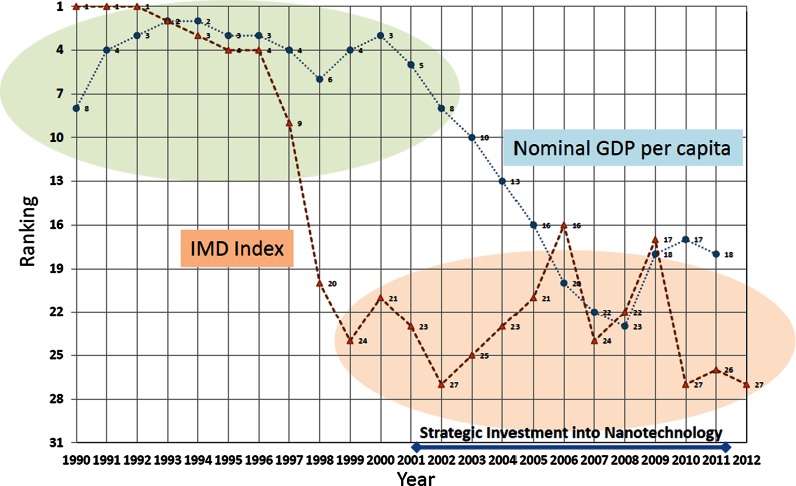
Japan’s international competitiveness

According to research by Switzerland’s International Institute for Management Development (IMD), Japan’s international productivity has continued to decline since 1996, based on indicators that cover economic performance, business efficiency, government efficiency, and the level of infrastructure development (IMD 2012). Science and technology generate new ideas and concepts, and have an impact on a country’s international competitiveness by creating new industries. Examining indicators such as the numbers of academic papers published by Japan (Negishi 2011) and intellectual property patents (JPO 2012), it is clear that the potential for Japan’s science and technology R&D is not low. However, the Japanese economy, which should be supporting the high R&D potential of science and technology, has been unable to escape deflationary economic conditions for more than twenty years. This declining curve did not change for the better even during the years 2001–2011, when Japan invested intensively in nanotechnology research and development. Because a decline in international competitiveness is reflected in the economy, Japan’s nominal GDP has also continued to decline, with a lag of a few years behind the downward curve of the IMD’s international competitiveness index. Japan’s considerable R&D investment has not yet led to economic stimulation from the new industries one would have expected to see arising from the new knowledge creation (Fig. 5).

This situation suggests that governmental policy-based innovation support initiatives, such as “seeds needs matching” (matchmaking between sources and potential users of new technologies) and industry-academia collaboration programs, did not actually help the recovery of the economy or even improve international competiveness. As is clear from the number of papers published, it was not a decline in R&D potential that caused a decline in international competitiveness, but that the R&D potential was not adequately utilized, and did not lead to a recovery in international competitiveness. It was the large gap between the two that was the problem.

The ability to make the most of R&D potential is based on proper science and technology management. Emerging sciences and technologies with any promise to narrow the gap between R&D potential and international competitiveness, however, have no choice but to move forward in R&D and applications, even without adequately established technology management frameworks in place such as risk assessment and industrial standardization. Meanwhile, for R&D outcomes to be utilized to create a sustainable society, it is essential to have a deeper understanding of societal needs and public expectations for emerging sciences and technologies. By advancing R&D that has involved public engagement by initiatives such as these at Osaka University, it is possible to close the gap that exists between R&D potential and international competitiveness. This paper introduced the educational program implemented by Osaka University’s Institute for NanoScience Design. It is a unique example in Japan in the area of capacity building for science and technology. These issues of public engagement should be integrated into capacity building programs in every science and engineering university. This kind of initiative for capacity building is not the sort of effort that can lead to visible outcomes in the short term. With the long-term view, however, these kinds of humble initiatives have the potential to help a recovery of the international competitiveness of Japanese industry, and even the country’s economic power.

## Remarks

This paper introduced a unique initiative for any Japanese university, the “Public Engagement and Nanotechnology” under Osaka University’s Nanotechnology Advanced Interdisciplinary Education, Research, and Training Program, which is being offered at the Institute for NanoScience Design (INSD). Only a few and limited programs are offered other than INSD. For example, Hokkaido University offers educational program to foster skills in science communication (CoSTEP), and CoSTEP held a workshop related to public engagement issues in nanotechnology R&D several years ago (CoSTEP 2007, 2014). The program offered by Osaka University is the only program which constantly provides courses on public engagement in nanotechnology R&D. This is the first multidisciplinary initiative toward issues of public engagement about nanotechnology , which have arisen from R&D, and provides many meaningful lessons for R&D of new technologies that will emerge in the future. Osaka University commences Graduate-level Career Development Programs for Nano Science and Nanotechnology program for 2014 academic year under the renewed government funding. The new program is “Saturdays Intensive Course,” “Public Engagement in Nanotechnology A” which delivers “Nanomaterials and EHS issues,” “Intellectual Property Management”, “Why, How, and to Whom Communicate” etc. (Itoh 2014). We believe that what Osaka University has learned based on its pioneering efforts should be replicated elsewhere in Japanese university education through government policy support, in order to promote responsible R&D in the future for emerging sciences and technologies.
